# Relationship of serum GDF11 levels with bone mineral density and bone turnover markers in postmenopausal Chinese women

**DOI:** 10.1038/boneres.2016.12

**Published:** 2016-06-21

**Authors:** Yusi Chen, Qi Guo, Min Zhang, Shumin Song, Tonggui Quan, Tiepeng Zhao, Hongliang Li, Lijuan Guo, Tiejian Jiang, Guangwei Wang

**Affiliations:** 1 Institute of Endocrinology and Metabolism, The Second Xiangya Hospital of Central South University, Changsha, China; 2 Hunan University of Medicine, Huaihua, China; 3 Department of Endocrinology, The Xiangya Hospital of Central South University, Changsha, China

## Abstract

Growth differentiation factor 11 (GDF11) is an important circulating factor that regulates aging. However, the role of GDF11 in bone metabolism remains unclear. The present study was undertaken to investigate the relationship between serum GDF11 level, bone mass, and bone turnover markers in postmenopausal Chinese women. Serum GDF11 level, bone turnover biochemical markers, and bone mineral density (BMD) were determined in 169 postmenopausal Chinese women (47–78 years old). GDF11 serum levels increased with aging. There were negative correlations between GDF11 and BMD at the various skeletal sites. After adjusting for age and body mass index (BMI), the correlations remained statistically significant. In the multiple linear stepwise regression analysis, age or years since menopause, BMI, GDF11, and estradiol were independent predictors of BMD. A significant negative correlation between GDF11 and bone alkaline phosphatase (BAP) was identified and remained significant after adjusting for age and BMI. No significant correlation was noted between cross-linked N-telopeptides of type I collagen (NTX) and GDF11. In conclusion, GDF11 is an independent negative predictor of BMD and correlates with a biomarker of bone formation, BAP, in postmenopausal Chinese women. GDF11 potentially exerts a negative effect on bone mass by regulating bone formation.

## Introduction

Osteoporosis, a classical age-related disease, is more common in women than in men.^1,2^ Osteoporosis affects approximately half of women over the age of 75 years. During the process of aging, estrogen deficiency, oxidative stress and lower IGF-1 level induce bone loss and osteoporosis.^1–5^

Recently, GDF11 was identified as a circulating age-associated factor that reverses age-related cardiac hypertrophy and skeletal muscle dysfunction and rejuvenates the aging neurodegenerative and neurovascular brain in mice.^6–8^ GDF11 is a member of the TGF-β super-family that circulates in the bloodstream of mammals.^9–11^ Some studies have shown that serum GDF11 levels in mice decreased during aging.^6–8^ By contrast, Egerman *et al.*^12^ showed that GDF11 increases with age in rats and humans and has a negative effect on muscle regeneration and satellite cell expansion in mice. Although the results regarding GDF11 are contradictory, it is certain that GDF11 has important roles in age-related diseases, including osteoporosis.

A recent study showed that GDF11 is a protective factor for osteoblastogenesis by inhibiting the activity of peroxisome proliferator-activated receptorγ (PPAR-γ).^13^ However, Li *et al.*^14^ demonstrated that antagonism of GDF11 activity in the transgenic mice promotes osteoblast activities and bone formation. Thus, the role of GDF11 in bone metabolism remains unclear.

The present study was undertaken to elucidate the relationship of serum GDF11 levels with bone mineral density (BMD) and bone turnover markers in postmenopausal Chinese women.

## Materials and methods

### Subjects

The study population included 169 healthy postmenopausal women (aged 47–78 years) selected randomly from Changsha (China) and its surrounding area. The mean duration from onset of menopause was 7.7±6.4 years with a median menopausal duration of 6 (range, 1–32) years. All subjects were screened using a detailed questionnaire, and a detailed medical history and physical examination was performed. Body height and weight were measured using a stadiometer and a standardized balance-beam scale, respectively.

Subjects were excluded from the study if they had conditions affecting bone metabolism, such as diseases of the kidney, liver, parathyroid, thyroid, diabetes mellitus, oligomenorrhea, menopause before the age of 40 years, hyperprolactinemia, oophorectomy, rheumatoid arthritis, ankylosing spondylitis, malabsorption syndromes, malignant tumors, or previous pathological fractures. Subjects were also excluded if they were taking glucocorticoids, estrogens, thyroid hormone, fluoride, bisphosphonate, calcitonin, thiazide, diuretics, barbiturates, or anti-seizure medications.

The study was approved by the Ethical Committee of Xiangya Hospital of Central South University, and all the participants provided written consent to participate.

### Serum GDF11 measurement

Blood samples were collected between 7:00 A.M. and 9:00 A.M. after fasting overnight and the samples were allowed to clot. Thereafter, the samples were centrifuged, divided into aliquots, and stored at −70 °C until assayed.

To minimize potential cross-reactivity with GDF8, we measured serum GDF11 concentration with a sensitive enzyme-linked immunosorbent assay (ELISA) kit according to Egerman’s^12^ methods. High bind 96-well multi-array plates were coated with 50 μL per well of 2 mg·mL^−1^ mouse anti-human GDF11 (R&D Systems, clone 743833) in phosphate-buffered saline (PBS) overnight at 4 °C. After washing with PBST, 300 μL of PBS with 3% bovine serum albumin (BSA) were added to each well and incubated with shaking at room temperature for 1–2 h. The samples were then washed with PBST. An eight-point calibration curve was prepared using three-fold dilutions, starting with a prepared sample of 100 ng·mL^−1^ recombinant human GDF11 in PBS with 1% BSA. PBS with 1% BSA was used as a blank. Samples were diluted 1:2 in PBS with 1% BSA. Fifty microliters of the calibration curve dilutions or samples were added in duplicate to wells and incubated with shaking for 3 h at room temperature. Following a PBST wash, 50 μL of a ruthenium-labeled antibody of mouse anti-human GDF11 (clone 743833) diluted to 4 mg·mL^−1^ in PBS with 1% BSA were added to each well and allowed to incubate in the dark with shaking for 2 h at room temperature. After an additional PBST wash step and the addition of 150 μL per well 2× MSD reader buffer with surfactant (4× MSD reader buffer with surfactant diluted 1:2 with distilled water), the plates were read using a μ-Quant Universal Microplate Spectrophotometer (Bio-Tek Instruments, Highland Park,Winooski, VT, USA). Sample concentrations were determined using a four-parameter logistic equation and 1/*Y*^2^ weighting. The coefficient of variation was <10% for GDF11.

### BMD measurements

BMD was measured by dual-energy X-ray absorptiometry using a Lunardevice (Lunar DPX IQ, MA, USA) as previously described.^15,16^ The following body parts were scanned: the lumbar spine (L1–L4) and the left hip, including the femoral neck (FN) and total hip (Hip). All BMD results are expressed in g·cm^−2^.

The precision of the dual-energy X-ray absorptiometry was evaluated over three repeated measurements of different BMD values in 22 subjects, and a mean coefficient of variation (CV) for all regions of 0.75%±0.21% (mean±s.d.; range: 0.47%–1.02%) was noted. A control spine phantom scan was performed daily with a long-term (more than 10 years) CV of 0.31%–0.43%, and the root-mean-square coefficient of variation was 0.35%.

### Hormone assays

Serum estradiol levels were determined using a competitive immunoassay, which uses direct chemiluminescence (Automated Chemiluminescence System 180 Estradiol-6 Assay, Bayer HealthCare LLC Diagnostics, Tarrytown, NY, USA). In addition, 25-hydroxyvitamin D was measured using ELISA kits (Biomedica Medizinprodukte GmbH & Co KG, Vienna, Austria). Serum intact parathyroid hormone (PTH) was measured using ELISA kits (Diagnostic System, Webster, TX, USA). The coefficient of variation was <10% for estradiol, 25-hydroxyvitamin D and PTH.

### Bone turnover markers measurement

The serum concentration of BAP, as the marker of bone formation, was measured using ELISA kits (BAP from Metra™ BAP EIA kit, Quidel Corporation, San Diego, CA, USA). As a marker of bone resorption, serum NTX was measured using ELISA kits (Osteomark, Ostex, Seattle, WA, USA). The coefficient of variation was <10% for BAP.

### Statistical analysis

SPSS 18.0 was used for the statistical analyses. The results are provided as the mean and standard deviation (mean±s.d.). The correlations between GDF11, BMD, and bone turnover markers were calculated using Pearson’s correlation analysis and partial correlation analysis. Multivariate linear stepwise analysis (forward selection) was performed to determine whether the level of variance in BMD at various skeletal regions could be explained by age, years since menopause, BMI, smoking habits, estradiol, 25-hydroxyvitamin D, PTH, and GDF11.

## Results

### Characteristics of the subjects

A total of 169 postmenopausal women were studied. Characteristics of the study participants regarding clinical, biochemical, demographic, and anthropometric characteristics are presented in Table 1.

### Correlation between GDF11 and age

**Figure 1:**
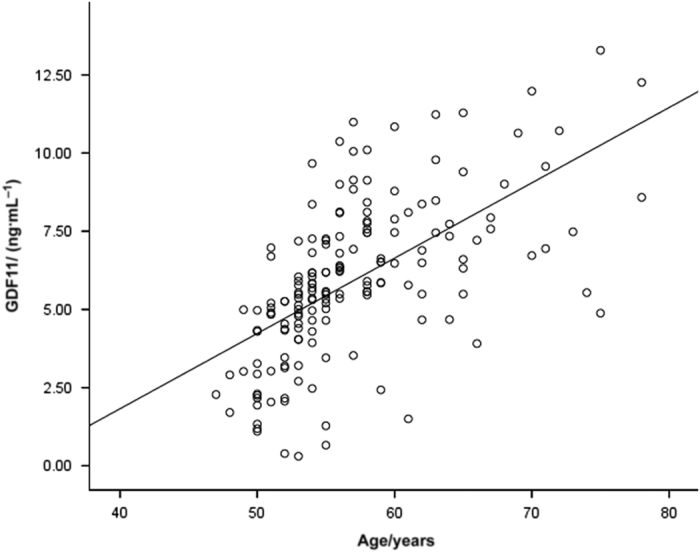
GDF11 serum levels increase with aging. GDF11 positively correlates with age (*r*=0.601, *P*<0.01). Scatter plot of serum GDF11 concentration versus age.

As shown in Figure 1, GDF11 serum levels increased with age. A positive correlation was noted between serum GDF11 and age (*r*=0.601, *P*<0.01).

### Relationships between GDF11 and BMD

Table 2 revealed significant negative correlations between GDF11 and BMD at the lumbar spine, total hip, and FN, and these results still remained significant after adjusting for age and BMI.

The parameters identified as significant and independent determinant factors of BMD by multiple linear regression analysis are presented in Table 3. The dependent variables were BMD at the lumbar spine, total hip and FN. The independent variables were entered in the regression analysis models, including age, years since menopause, BMI, smoking habit, serum 25-hydroxyvitamin D, PTH, estradiol, and GDF11. In the model with lumbar spine BMD as the dependent variable, the significantly independent variables were age, estradiol, and BMI, and these variables explained 20.9% of BMD variance (*R*^2^=0.209). In the model with total hip BMD as the dependent variable, the significantly independent variables were years since menopause, BMI, GDF11 and estradiol, and these variables explained 27.4% of BMD variance (*R*^2^=0.274). In the model with FN BMD as the dependent variable, the significantly independent variables were age, GDF11, estradiol, and BMI, and these variables explained 16.9% of BMD variance (*R*^2^=0.169). These results indicate that GDF11 was the negative significant influencing factor for BMD of the total hip and FN.

### Associations between GDF11 and bone turnover markers

Serum BAP as a bone formation marker and serum NTX as a bone resorption marker were assayed. As shown in Table 4, a significant negative correlation was noted between GDF11 and BAP. The correlation remained significant after adjusting for age and BMI. No significant correlation was noted between GDF11 and NTX.

## Discussion

Osteoporosis is an age-related disease.^1,17^ Sex hormone deficiencies, endogenous hyperglucocorticoidism, secondary parathyroidism, and oxidative stress induce age-related osteoporosis.^17,18^ A number of factors have an effect on the process of bone formation. Circulating cytokines also have important roles. Xian *et al.*^5^ demonstrated that IGF-1 in bone marrow maintains bone mass by activation of mTOR in BMSCs, and bone marrow IGF-1 concentrations were reduced in age-related osteoporosis. Recently, GDF11 was identified as a circulating age-associated factor that reverses age-related cardiac hypertrophy and skeletal muscle dysfunction, and rejuvenates the aging neurodegenerative and neurovascular brain in mice.^6–8^ However, Egerman *et al.*^12^ showed that GDF11 increases with age in rats and humans and has a negative effect on muscle regeneration and satellite cell expansion in mice. Research regarding the role of GDF11 during aging remains contradictory. Our study showed that serum GDF11 levels positively correlated and increased with age.

Our results were consistent with Egerman’s study, and found that serum GDF11 concentration gradually increased with age. Egerman *et al.*^12^ developed aGDF11-specific immunoassay to detect the GDF11 concentration in serum of young and aged individuals and revealed a trend toward increased GDF11 levels in serum of aged individuals. The GDF11 antibody used in the previous study exhibited cross-reactivity with GDF8 and did not correctly measure the GDF11 concentration. Our results were not consistent with the recent study by Zhang *et al.*,^13^ who showed that GDF11 levels significantly decreased in the serum of aging mice and human. They also showed that serum GDF11 levels decreased in women with osteoporosis compared with normal patients.^13^ One possible explanation is that the antibody to GDF11 used by Zhang’ study was not specific and that GDF8 was also detected. Our study is consistent with the results obtained by Egerman and showed that GDF11 levels increase with age.

Next, we investigated the relationship between GDF11 and BMD. Our results showed that GDF11 correlated negatively with BMD and was the negative determinant of total hip and FN BMD in postmenopausal women. Our study suggested that GDF11 exerts a negative effect on bone mass. To date, researchers report opposite conclusions about the function of GDF11; however, several studies support our results. Myostatin null mice exhibit increased osteoblast activity and increased bone mass.^19–21^ The GDF11 amino-acid sequence is 90% identical to myostatin (GDF8).^22,23^ Moreover, studies demonstrated that GDF11 and myostatin both induce SMAD2/3 phosphorylation and regulate identical downstream signaling.^9–12^ GDF11 and myostatin similarly inhibit muscle regeneration and decrease satellite cell expansion in mice.^12,24^ Together, these results suggest that GDF11 and myostatin have a similar role in bone metabolism. Li *et al.*^14^ generated transgenic mice with skeleton-specific overexpression of GDF11 propeptide cDNA, which antagonized GDF11 activity in osteoblasts. These transgenic mice have increased osteoblast activity and enhanced bone formation. Li *et al.*^14^ suggested that inhibition of GDF11 activity stimulates bone formation by increasing the osteoblast function.

Furthermore, we found that GDF11 negatively correlates with the bone formation biochemical marker, BAP. This finding supports the hypothesis that GDF11 may induce bone loss by inhibiting bone formation.

In conclusion, the present study has provided evidence that GDF11 is an independent negative predictor of total hip and FN BMD in postmenopausal Chinese women and demonstrates a negative correlation with a bone formation biochemical marker. Taken together, these data demonstrated that the increased GDF11 levels in serum with aging predicted bone loss, suggesting a potential role for GDF11 in regulating bone formation. The mechanism should be further clarified.

## Figures and Tables

**Table 1:** Subject characteristics and bone and biochemical parameters of 169 postmenopausal women

Detected parameters	Postmenopausal women
Age/years	57.0±6.3
Years since menopause	7.7±6.4
Height/cm	155.6±5.0
Body weight/kg	53.5±4.8
BMI/(kg·m^−2^)	22.1±1.4
BMD (g·cm^−2^)	
Lumbar spine	0.884±0.135
Total hip	0.837±0.112
Femoral neck	0.734±0.098
GDF11/(ng·mL^−1^)	5.92±2.52
Estradiol/(pg·mL^−1^)	155.8±95.7
25-hydroxyvitamin D/(ng·mL^−1^)	33.0±8.0
PTH/(pg·mL^−1^)	41.1±24.2
BAP/(U·L^−1^)	28.2±11.2
NTX/(nmol·L^−1^)	18.8±9.9

BMD, bone mineral density; BMI, body mass index; GDF11, growth differentiation factor 11; PTH, parathyroid hormone; BAP, bone alkaline phosphatase; NTX, cross-linked N-telopeptides of type I collagen.

Values are presented as mean±s.d.

**Table 2:** Correlation coefficients of serum GDF11 and BMD

BMD in different bone	GDF11	
	Unadjusted	Age and BMI adjusted
Lumbar spine BMD	−0.271*	−0.153*
Total hip BMD	−0.319*	−0.152*
Femoral neck BMD	−0.272*	−0.152*

BMD, bone mineral density; BMI, body mass index; GDF11, growth differentiation factor 11.

Pearson’s correlation coefficients and partial correlation coefficients after adjusting for age and BMI are presented.

*P<0.05.

**Table 3:** Parameters identified as significant and independent predictors of BMD

Parameters	Lumbar spine BMD (*R*^2^=0.209)		Total hip BMD (*R*^2^=0.274)		Femoral neck BMD (*R*^2^=0.169)	
	β-coefficient	*P*-values	β-coefficient	*P*-values	β-coefficient	*P*-values
Age	—	—	—	—	−0.203	0.025
Years since menopause	−0.363	0.000	−0.340	0.000	—	—
GDF11	—	—	−0.197	0.014	−0.183	0.044
BMI	0.176	0.014	0.266	0.000	0.178	0.016
Estradiol	0.227	0.001	0.147	0.030	0.180	0.013

BMD, bone mineral density; GDF11, growth differentiation factor 11.

**Table 4:** Correlation of serum GDF11 levels with bone turnover markers

Bone turnover markers	GDF11	
	Unadjusted	Age and BMI adjusted
BAP	−0.306*	−0.258*
NTX	0.024	0.081

BAP, bone alkaline phosphatase; BMI, body mass index; GDF11, Growth Differentiation Factor 11; NTX, Cross-linked N-telopeptides of type I collagen.

Pearson’s correlation coefficients and partial correlation coefficients after adjusting for age and BMI are presented.

*P<0.05.
